# α-Mangostin Promotes In Vitro and In Vivo Degradation of Androgen Receptor and AR-V7 Splice Variant in Prostate Cancer Cells

**DOI:** 10.3390/cancers15072118

**Published:** 2023-04-01

**Authors:** Mirielle C. Nauman, Jong Hoon Won, Sakina M. Petiwala, Bhaskar Vemu, Hyun Lee, Maria Sverdlov, Jeremy J. Johnson

**Affiliations:** 1 Department of Pharmaceutical Sciences, College of Pharmacy, University of Illinois at Chicago, Chicago, IL 60612, USA; 2 Department of Pharmacy Practice, College of Pharmacy, University of Illinois at Chicago, Chicago, IL 60612, USA; 3 Biophysics Core at Research Resource Center, University of Illinois at Chicago, Chicago, IL 60607, USA; 4 Research Histology and Tissue Imaging Core, University of Illinois at Chicago, Chicago, IL 60612, USA

**Keywords:** prostate cancer, mangosteen, α-mangostin, androgen receptor, AR-V7, BiP, GRP78, xanthone, *Garcinia mangostana*

## Abstract

**Simple Summary:**

Castration-resistant prostate cancer often develops in response to continued drug treatment and is characterized by mutations in the androgen receptor. Commonly used anti-androgen drugs are ineffective at targeting these cells; interestingly, our study evaluated a compound from *Garcinia mangostana* that has activity against castration-resistant prostate cancer cells. Our data shows that this compound effectively decreases cell viability and degrades the androgen receptor, regardless of mutations present in the androgen receptor. We suggest that this may be achieved through the activation of a cell stress chaperone protein, BiP, which our compound both binds to and promotes binding with the androgen receptor. Finally, this compound has in vivo anticancer activity and decreases mutant androgen receptor expression in vivo as well. Novel compounds that can degrade mutant androgen receptors present an opportunity to target castration-resistant prostate cancer cells through a unique mechanism.

**Abstract:**

A major limitation of current prostate cancer pharmacotherapy approaches is the inability of these compounds to target androgen receptor variants or mutants that develop during prostate cancer progression. The demand for novel therapeutics to prevent, slow, and treat prostate cancer is significant because FDA approved anti-androgens are associated with adverse events and can eventually drive drug-resistant prostate cancer. This study evaluated α-mangostin for its novel ability to degrade the androgen receptor and androgen receptor variants. α-Mangostin is one of more than 70 isoprenylated xanthones isolated from *Garcinia mangostana* that we have been evaluating for their anticancer potential. Prostate cancer cells treated with α-mangostin exhibited decreased levels of wild-type and mutated androgen receptors. Immunoblot, immunoprecipitation, and transfection experiments demonstrated that the androgen receptor was ubiquitinated and subsequently degraded via the proteasome, which we hypothesize occurs with the assistance of BiP, an ER chaperone protein that we have shown to associate with the androgen receptor. We also evaluated α-mangostin for its antitumor activity and promotion of androgen receptor degradation in vivo. In summary, our study demonstrates that androgen receptor degradation occurs through the novel activation of BiP and suggests a new therapeutic approach for prostate cancer.

## 1. Introduction

Approximately 1,200,000 men are diagnosed with prostate cancer (PCa), and 300,000 men worldwide die from PCa each year, accounting for 4% of all male cancer-related deaths and making PCa the second most common cancer in men [1,2]. Though PCa tumors are relatively slow growing and there is a high correlation between lifestyle choices/other risk factors and PCa mortality, the demand for effective PCa pharmacotherapies and chemoprevention agents is high, considering the large population that PCa affects and that current drugs are not always effective [3,4]. PCa is predominantly hormone-responsive and is largely directed by the androgen receptor (AR), which has been detected in almost all prostate cell lines and directs androgen signaling. AR is a nuclear receptor that maintains the proliferation of prostate cells by directing the transcription of more than 800 different downstream genes, many of which are involved in homeostasis, cell cycle regulation, the regulation of energy metabolism, signal transduction, and protein trafficking [5,6]. Accordingly, PCa cells often manipulate AR and promote carcinogenesis through changes in androgen biosynthesis, aberrant androgen signaling, AR overexpression, and point mutations and/or splice variants within the AR gene [7,8]. Many of these changes present opportunities for resistance to occur; thus, much effort has gone into studying AR and designing drugs that can target AR.

One class of currently used PCa drugs, anti-androgens, are competitive AR antagonists that target the androgen signaling pathway by competing with dihydrotestosterone and thereby blocking expression of genes involved in cancer promotion. The anti-androgens flutamide, bicalutamide, and enzalutamide show efficacy in early-stage and localized PCa cases but are less effective in later-stage cases or when AR is mutated [9,10]. Molecular resistance to long-term usage of anti-androgens is evidenced in AR containing point mutations in their ligand binding domain (e.g., F876L and T878A) or in splice variants such as AR variant 7 (AR-V7) [11,12]. AR-V7 has been shown to be constitutively activated in drug-resistant, or castration-resistant PCa (CRPC), and significantly differs from wild-type AR due to its shortened structure and lack of a ligand binding domain, making it difficult to target with small molecules [13]. One interesting solution to this has been to target the N-terminal domain of AR, and one such compound has reached Phase I clinical trials [14,15,16]. Promoting the degradation of AR and AR-V7 rather than targeting the proteins themselves, represents a novel strategy to overcome CRPC.

Recent studies have reported that chemical compounds isolated from the purple mangosteen fruit (*Garcinia mangostana*) have anticancer activity in colon, breast, lung, cervical, and prostate cancer cells, as well as leukemia and melanoma cells [17,18,19,20,21]. Our group has been studying xanthones for their in vitro and in vivo anticancer activity, and our data suggests that xanthones promote apoptosis in PCa cells, degrade AR, and slow prostate tumorigenesis in mice [22,23,24,25,26,27,28,29]. This study was undertaken to test our hypothesis that α-mangostin, the most abundant mangosteen xanthone, promotes the ubiquitination and degradation of AR and AR-V7 and that this is achieved through the activation of BiP, an endoplasmic reticulum (ER) cell stress and chaperone protein.

## 2. Materials and Methods

### 2.1. Materials

α-Mangostin (purity 92.2%, CAS: 6147-11-1) was purchased from Chromadex (Los Angeles, CA, USA). 3-(4,5-dimethylthiazol-2-yl)-2,5-diphenyltetrazolium bromide (MTT, purity 98%, CAS: 298-93-1) was purchased from Acros Organics (Waltham, MA, USA). Bicalutamide (purity 98%, CAS: 90357-06-5) was purchased from Adooq Bioscience (Irvine, CA, USA). (S)-MG132 (purity 98%, CAS: 133407-82-6) and PD169316 (purity 98%, CAS: 152121-53-4) were purchased from Cayman Chemical (Ann Arbor, MI, USA). Antibodies against androgen receptor (5153), ubiquitin (3936), β-actin (4970), GAPDH (2118), Lamin A/C (4777), BiP (3177), and PERK (5683) were purchased from Cell Signaling Technologies (Beverly, MA, USA). Antibodies against IgG (66931), GRP78 (166490), androgen receptor (441), and GST (138) were purchased from Santa Cruz Biotechnologies (Dallas, TX, USA). AR-V7 antibody (AG10008) was purchased from Precision Antibody (Columbia, MD, USA). Cleaved caspase-3 (Asp175) and AR-V7 ELISA kits were purchased from Cell Signaling Technologies. A human PSA ELISA kit was purchased from Anogen (Mississauga, ON, Canada).

LNCaP, PC-3, 22Rν1, and VCaP cells were purchased from ATCC (Manassas, VA) and were cultured in 37 °C incubators containing 5% carbon dioxide. LNCaP and 22Rν1 cells were maintained in RPMI-1640 media (Gibco, Waltham, MA, USA) supplemented with 10% fetal bovine serum (Bio-Techne, Minneapolis, MN, USA) and 1% penicillin/streptomycin (Corning, Corning, NY, USA), PC-3 cells were cultured in Ham’s F-12K media (Gibco) supplemented with 10% fetal bovine serum and 1% penicillin/streptomycin, and VCaP cells were maintained in DMEM media (Gibco) supplemented with 10% fetal bovine serum and 1% penicillin/streptomycin.

### 2.2. MTT Assays

LNCaP or 22Rν1 cells (3 × 10^5^) and VCaP cells (5 × 10^5^) were plated in black 96-well plates and incubated. After 24 (22Rν1) or 48 (LNCaP, VCaP) hours, the media was removed and replaced with media containing α-mangostin at concentrations ranging from 0–100 µM. The DMSO vehicle level was kept under 0.01%. Plates were incubated with treatments for 48 h. Media containing treatments were removed and replaced with media containing MTT reagent at 20 mg/mL. Plates with MTT were wrapped in foil and incubated for 4 h. Finally, media was carefully aspirated and replaced with sterile DMSO; plates were put on a room temperature shaker for 10 min while wrapped in foil. Absorbance readings were taken at 570 nm in a multi-well plate reader, and cell viability/IC_50_s were calculated. All incubations were done in a 4 °C incubator containing 5% CO_2_.

### 2.3. ELISA Assays

2–3 × 10^5^ cells were plated and incubated for 24 h. Media was removed and replaced with media containing 0–10 µM of α-mangostin for 24 h. Cell lysates were extracted with freshly prepared lysis buffer. 30 µg of protein from each treatment was used in either a pre-treated cleaved caspase-3 enzyme-linked immunosorbent assay (ELISA) plate or a pre-treated AR-V7 ELISA plate, and washes and incubations were performed according to the manufacturer’s instructions. Absorbance readings were taken at 450 nm.

Similarly, 50 μL of mouse plasma or prostate specific antigen (PSA) standard were incubated in a pre-treated PSA ELISA plate. Washes and incubations were performed according to the manufacturer’s instructions. Absorbance readings were taken at 450 nm.

### 2.4. Western Blotting

To isolate protein from whole cell lysates, treated cells were washed with ice-cold phosphate-buffered saline (PBS). Cell lysis buffer (Cell Signaling Technologies) supplemented with protease and phosphatase inhibitors was added to culture plates, and the plates were scraped to lyse the cells. Homogenates were collected, vortexed, and passed through a 25-gauge needle. Lysates were spun for 30 min at 4° at 13,000 RPM. The resulting supernatants were collected, and protein concentrations were estimated using a BCA kit (Pierce, Waltham, MA, USA) according to the manufacturer’s instructions.

To isolate protein from tumor tissue, 20 mg of each tumor tissue sample was mixed 1:20 with T-PER reagent (Thermo Scientific, Waltham, MA, USA) supplemented with protease inhibitor and homogenized.

Cell and tumor protein extracts were run following a Western blot protocol, as we described previously [30]. Briefly, equal amounts of protein were loaded into 4–15% pre-cast gels (Bio-Rad, Hercules, CA, USA). The gels were transferred onto nitrocellulose membranes, and the membranes were then blocked for 1 h at room temperature. After blocking, membranes were cut horizontally into strips in order to evaluate multiple proteins of interest, and membrane strips were then incubated with their respective primary antibodies overnight at 4 °C with shaking. Membrane strips were washed, incubated with secondary antibody at room temperature for 2 h, and then washed again before being incubated with a glow substrate and then exposed in an Invitrogen iBright CL1500 imager (Waltham, MA, USA). ImageJ software was used to quantify protein expression.

### 2.5. Quantitative PCR (qPCR)

22Rν1 cells (1 × 10^6^) were plated in 6-well plates and incubated for 24 h. α-Mangostin was added to the plates at either 10 or 20 μM concentrations for 24 h. RNA was then extracted from the cells using an RNAqueous™-4PCR Total RNA Isolation Kit (#AM1914, Invitrogen, Waltham, MA, USA) by following the recommended protocol. RNA was resuspended in nuclease-free water.

RNA was extracted from tumor tissues using an RNeasy PowerLyzer Tissue & Cells kit (#15055, Qiagen, Germantown, MD, USA), according to the manufacturer’s instructions, and RNA was resuspended in nuclease-free water.

cDNA was made with the iScript™ cDNA Synthesis kit (#1708890, Bio-Rad), according to the manufacturer’s instructions. qPCR was run using PowerUp™ SYBR™ Green Master Mix (Thermo Fisher, Waltham, MA, USA) according to the manufacturer’s protocol. All primers were designed using the NCBI-Primer Blast tool and ordered from Integrated DNA Technologies (Coralville, IA, USA).

### 2.6. Immunoprecipitations

Immunoprecipitations were performed using total protein from treated whole cell lysates that were collected as described above. Specific antibodies were incubated with magnetic beads (Dynabeads™ G, Invitrogen) on an end-over-end shaker. The antibody-bead complex was crosslinked using BS3 (Thermo Scientific). Cell lysates (1 mg) were then incubated with the beads on an end-over-end shaker, and protein complexes were eluted according to the manufacturer’s instructions. Protein complexes were denatured and analyzed for the indicated proteins by Western blot.

### 2.7. Plasmid Transfections

PC-3 cells (5 × 10^5^) were plated and incubated for 24 h. The cells were transfected with 0.5–1 µg of the indicated ΔAR plasmid using *Trans*IT^®^-2020 transfection reagent (Mirus, Madison, WI, USA) and MEM media (Gibco) by following the manufacturer’s instructions. Transfected cells were incubated for 24 h and then treated with 0–20 µM α-mangostin for another 24 h. Normal cell lysis procedures were then followed.

Similarly, 4 × 10^6^ 22Rν1 cells were plated and incubated for 24 h. The cells were transfected with either His-GST-BiP or BiPΔC41A (15 µg) plasmids following the same procedure. Transfected cells were incubated for 24 h and then treated with 0 or 10 α-mangostin for another 24 h. Normal cell lysis procedures were then followed.

### 2.8. Site Directed Mutagenesis

The NEBaseChanger™ online tool was used to create primers that were ordered from Integrated DNA Technologies. The Q5^®^ Site-Directed Mutagenesis kit (#E0554, New England Biolabs, Ipswich, MA, USA) was utilized with plasmids that contained DNA coding either for the androgen receptor (RC215316, Origene) or GRP78 (RC205859, Origene). Reagents and DNA were assembled in thin-walled PCR tubes, and 25 cycles of PCR were run with separate programs that were optimized for each experiment (changes in the annealing and extension steps were made based on the T_M_ of the primers used and the size of the plasmids used). This was followed by KLD treatment at room temperature for 10 min and transformation into *E. coli* cells.

### 2.9. Protein Purification

All plasmids were shuttled into the bacterial expression vector pEX-N-His-GST (PS100028, Origene) using T4 DNA Ligase (New England Biolabs) and AsiSI and MluI-HF restriction enzymes (New England Biolabs) before protein expression and purification. An overnight culture of transfected *E. coli* cells was added to 250 mL LB broth at a 1:100 dilution and then incubated for 3 h at 37 °C with shaking. Cells were induced with 1 mM IPTG (GoldBio, Olivette, MO, USA) for 2 h at 37 °C with shaking. Induced LB broth was centrifuged, and the resulting pellet was resuspended in B-PER (Pierce) and allowed to incubate at room temperature with shaking for 15 min. This mixture was centrifuged, and the resulting cell lysate was then used in the GST Spin Purification kit (#16108, Thermo Fisher), following the manufacturer’s instructions, to purify recombinant protein. Purified protein was concentrated using Amicon™ Centrifugal Filter units (MilliporeSigma™, Burlington, MA, USA), aliquoted and spiked with 10% glycerol, and flash frozen into stocks that were kept at −80 °C until use.

### 2.10. Surface Plasmon Resonance (SPR)

SPR analyses were conducted by the Biophysics Core at UIC. All runs were performed using a Biacore T200 instrument (Cytiva, Emeryville, CA, USA). Prior to immobilization on a NTA chip, the sensor chip surface was cleaned with 350 mM EDTA for 60 s and saturated with Ni^2+^ by injecting 0.5 mM NiCl_2_, followed by surface activation with 1-ethyl-3-(3-dimethylaminopropyl) carbodiimide hydrochloride (EDC)/N-hydroxy succinimide (NHS) mixture for 7 min at a 10 μL/min flow rate. Then purified BiP-N-His-GST protein was diluted to 30 μg/mL with a running buffer (10 mM HEPES, pH 7.4, 150 mM NaCl, 0.05% Tween-20) and injected to the prepared NTA surface for 7 min at 10 μL/min. An off-target reference protein was also immobilized on a separate channel of the same NTA chip in the same manner. Analytes were tested in triplicate at concentrations ranging from 0–25 μM (3-fold dilution) in a binding buffer containing 10 mM HEPES, pH 7.4, 150 mM NaCl, 0.05% Tween-20, 2 mM MgCl_2_, and 2% DMSO. Single cycle kinetics were used, and the runs were performed with 90 sec association and 600 s dissociation times at a flow rate of 30 μL/minute at 25 °C. The data were double referenced with a reference channel and zero concentration (2% DMSO) responses and fitted with a 1:1 Langmuir kinetic model using a Biacore Insight evaluation software. Steady-state affinity fittings were also done with binding responses during the equilibration phase, and the dissociation constants (*K_D_*) values were determined by fitting the data to a single rectangular hyperbolic curve Equation (1), where *y* is the response, *y_max_* is the maximum response, and *x* is the compound concentration.

(1) $$y=\frac{y_{max}\cdot x}{K_{D}+x}$$

### 2.11. In Vivo 22Rv1 Xenograft

Twenty-four male athymic (*nu*/*nu*) nude mice were purchased from Jackson Laboratory (Bar Harbor, Maine, USA) and were randomly divided into three groups, with eight animals in each group. Mice were housed in pathogen-free conditions with a 12 h light/12 h dark schedule and were fed an autoclaved AIN-76A diet ad libitum. At 7 weeks old, mice were injected with 1.25 × 10^6^ cells on each flank that were suspended 1:1 in Matrigel (Corning). Each group received treatments by oral gavage daily, starting the day of xenograft. One group received corn oil with 1% DMSO (control), another group received 3 mg/kg bicalutamide in corn oil with 1% DMSO, and another group received 50 mg/kg α-mangostin in corn oil with 1% DMSO. Body weights were recorded consistently throughout the study, and tumors were measured with electronic calipers. Animals were monitored daily and were consistently evaluated for health by the animal care staff. At the conclusion of the study, mice were dosed accordingly, submandibular blood collection was performed approximately 1 h after dosing, and mice then underwent CO_2_ asphyxiation per the approved protocol for euthanasia. All animal experiments were performed in accordance with the guidelines approved by the Animal Care and Use Committee of the University of Illinois at Chicago and under the approved protocol (#ACC-19-199).

### 2.12. Immunohistochemistry

Xenografts were fixed in 10% buffered formalin for 48 h at room temperature, processed through graded alcohols and xylene in the ASP 300S automated tissue processor (Leica Biosystems, Deer Park, IL, USA), and embedded in paraffin blocks. Tissue samples were sectioned at 5 µm and slides were baked for 1 h at 60 °C prior to H and E and IHC staining. H and E staining was carried out on the Autostainer XL (Leica Biosystems) following a preset protocol. For immunohistochemistry, slides were deparaffinized and stained on the BOND RX autostainer (Leica Biosystems) using the Mouse-on-Mouse Polymer IHC kit (#ab269452, Abcam, Waltham, MA, USA) and the BOND Research Detection System (Leica Biosystems). Briefly, tissue sections were subjected to antigen retrieval with BOND Epitope Retrieval Solution 2 (#AR9640, Leica Biosystems) for 40 min at 100 °C, incubated with Blocking Reagent for 15 min, and then incubated with anti-androgen receptor antibody at a 1:50 dilution for 1 h at RT. Slides were incubated with an HRP-polymer detector and developed with the Betazoid DAB Chromogen Kit (#BDB2004, Biocare Medical, Pacheco, CA, USA). Slides were stained with hematoxylin, followed by rehydration on the Autostainer XL,e and coverslipped with Surgipath Micromount Media (#3801730, Leica Biosystems).

### 2.13. Calculations and Statistical Analysis

Prism (GraphPad Software, San Diego, CA, USA) was used to calculate IC_50_ values using nonlinear regression analyses and to conduct one-way ANOVA or student’s *t*-test analyses. *p* values ≤ 0.05 were considered statistically significant.

## 3. Results

### 3.1. α-Mangostin Promotes Apoptosis and Inhibits Nuclear Translocation

**Figure 1:**
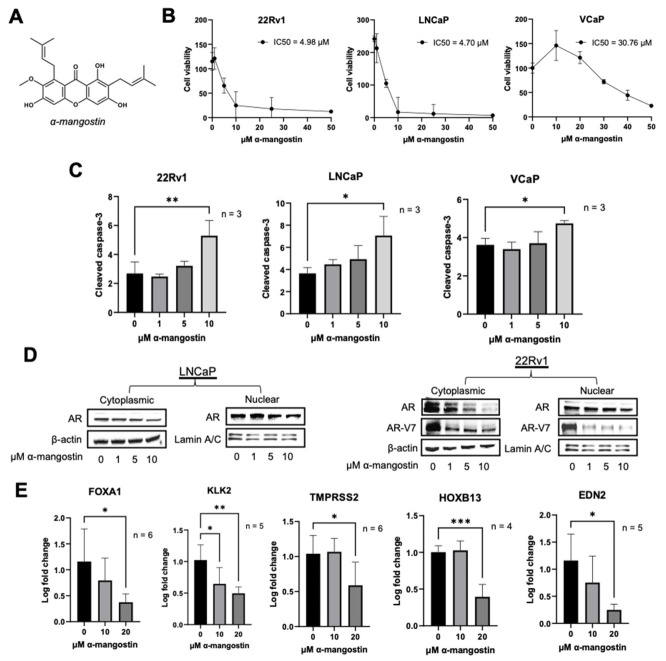
α-Mangostin promotes apoptosis and inhibits nuclear translocation. (**A**) Structure of α-mangostin. (**B**) Cells were treated with α-mangostin and cell viability was determined by MTT assays. (**C**) Cells were treated with 0–20 μM α-mangostin for 24 h and cleaved caspase-3 levels were analyzed by ELISA. 30 µg of protein from cell lysates were used. (**D**) Nuclear and cytoplasmic fractions from cells treated with α-mangostin for 24 h were analyzed for androgen receptor expression by Western blot. (**E**) AR- and AR-V7-regulated genes were analyzed by qPCR in 22Rν1 cells treated with α-mangostin for 24 h. * *p* ≤ 0.05, ** *p* ≤ 0.01, and *** *p* ≤ 0.001. The uncropped blots are shown in File S1.

The effect of α-mangostin on the viability of human PCa cells was evaluated in 22Rν1, LNCaP, and VCaP cell lines by MTT assays. α-Mangostin (Figure 1A) dose-dependently decreased PCa cell viability across all cell lines after 48 h of treatment with half maximal inhibitory concentrations (IC_50′_s) ranging from 5–30 μM (Figure 1B). Following treatment with α-mangostin (10 μM), there were statistically significant increases in cleaved caspase-3 levels in 22Rν1, LNCaP, and VCaP cells as determined by ELISAs, suggesting apoptosis (Figure 1C). Nuclear translocation of AR was evaluated by separating the nuclear and cytoplasmic fractions of LNCaP, 22Rν1, and VCaP cells treated with α-mangostin; the cytoplasmic and nuclear levels of both AR and AR-V7 decreased after α-mangostin treatments (Figure 1D and Figure S1). We evaluated the expression of AR and AR-V7 target genes FOXA1, KLK2, TMPRSS2, HOXB13, and EDN2 in 22Rν1 cells by qPCR. 22Rν1 was the most suitable cell line for this analysis as it expresses both AR and AR-V7 without any other significant alterations. There were significant decreases in the expressions of all five genes in cells treated with 20 μM α-mangostin (Figure 1E).

### 3.2. α-Mangostin Promotes Ubiquitination and Degradation of AR and AR-V7 via the Proteasome

**Figure 2:**
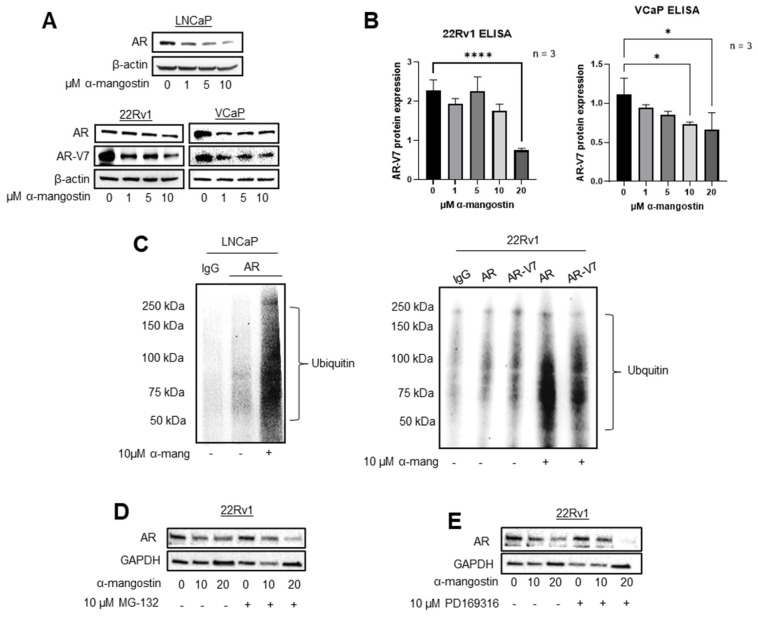
α-Mangostin promotes the ubiquitination and degradation of AR and AR-V7 via the proteasome. (**A**) AR and AR-V7 expression was evaluated in α-mangostin-treated PCa cells, including LNCaP, 22Rν1, and VCaP cells. (**B**) 22Rν1 and VCaP cells were treated with α-mangostin for 24 h and then whole cell lysates were evaluated for their expression of total AR-V7 through ELISA assays. (**C**) AR proteins from LNCaP and AR and AR-V7 proteins from 22Rν1 were immunoprecipitated with either anti-AR or anti-AR-V7 antibodies. Ubiquitinated AR and AR-V7 were analyzed by Western blot. (**D**) AR expression was analyzed after either individual or co-treatments of α-mangostin and/or MG-132. (**E**) AR expression was analyzed after either individual or co-treatments of α-mangostin and/or PD169316. * *p* ≤ 0.05 and **** *p* ≤ 0.0001. The uncropped blots are shown in File S1.

AR expression was evaluated in PCa cell lines treated with α-mangostin. After 24 h of treatment with α-mangostin, the expression of AR decreased dose-dependently, and was nearly undetectable in LNCaP cells at the 10 μM concentration. In 22Rν1 and VCaP cells, the expressions of AR and AR-V7 also decreased dose-dependently after α-mangostin treatment (Figure 2A). The decrease of AR-V7 specifically in response to α-mangostin treatments was also confirmed through ELISA assays and was statistically significant (Figure 2B). Using an immunoprecipitation assay, we observed that AR is ubiquitinated in LNCaP cells treated with α-mangostin and likewise, that AR and AR-V7 are both ubiquitinated in 22Rν1 cells treated with α-mangostin (Figure 2C). To confirm the involvement of the proteasome, the expression of AR was evaluated in 22Rν1 cells treated with either 10 μM α-mangostin, 1 μM MG-132, or 1 μM PD169316, synthetic proteasome inhibitors or activators (achieved through the inhibition of the p38 MAPK pathway) [31], respectively, or combination treatments of 10 μM α-mangostin + 1 μM MG-132 or 1 μM PD169316. When treated with α-mangostin and MG-132, AR expression still showed a slight decrease; however, this may be due to cell death caused by MG-132. When cells were treated with α-mangostin and PD169316, AR was degraded, and its expression was almost undetectable (Figure 2C,D).

### 3.3. α-Mangostin Decreases Mutant AR Expression and Promotes the Expression of BiP

**Figure 3:**
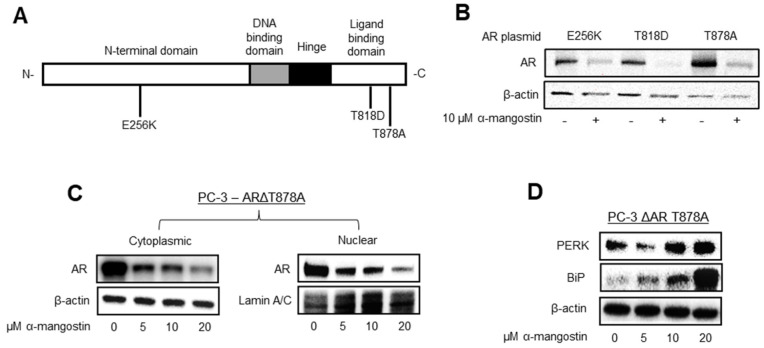
α-Mangostin decreases mutant AR expression and promotes the expression of BiP. (**A**) Structure of AR with single amino acid point mutations. (**B**) AR expression was evaluated in α-mangostin-treated PC-3 cells that had been transfected with 0.5–1 µg of ΔAR E256K, T818D, or T878A plasmid. (**C**) AR expression was evaluated in PC-3 cells treated with varying concentrations of α-mangostin that had been transfected with 0.5 µg of ΔAR T878A plasmid. (**C**) AR expression was evaluated in the nuclear and cytoplasmic fractions of cell lysates from α-mangostin-treated PC-3 cells transfected with 0.5 µg of ΔAR T878A plasmid. (**D**) Westerns of PERK and BiP expression in PC-3 cells transfected with ΔAR T878A cDNA after 24-h treatments. The uncropped blots are shown in File S1.

AR plasmids containing single point mutations, including E256K, T818D, and T878A (Figure 3A), were made using site-directed mutagenesis and transiently transfected into PC-3 cells. After α-mangostin treatments, the expression of mutant AR protein decreased in transfected cells (Figure 3B). Nuclear translocation of ΔAR-T878A was evaluated by separating the nuclear and cytoplasmic fractions of transiently transfected PC-3 cells treated with α-mangostin; the cytoplasmic and nuclear levels of ΔAR-T878A decreased after α-mangostin treatments (Figure 3C). The expressions of cell stress proteins PERK and BiP were found to increase in transiently transfected PC-3 cells treated with α-mangostin as evaluated by Western blots (Figure 3D).

### 3.4. α-Mangostin Increased BiP Expression, Promoted a Protein-Protein Interaction between AR and BiP, and Binds to BiP

**Figure 4:**
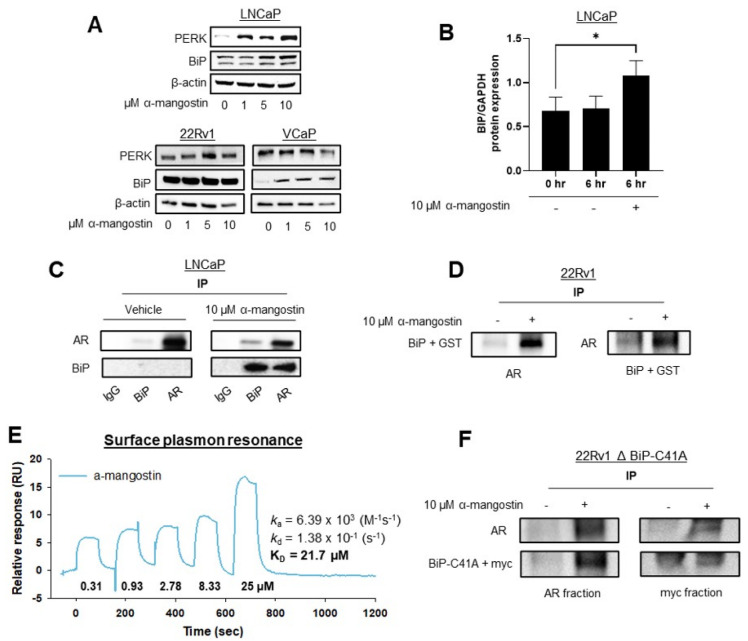
α-Mangostin increased BiP expression, promoted a protein-protein interaction between AR and BiP, and binds to BiP. (**A**) Westerns of PERK and BiP expression after 24-h treatments. (**B**) Quantification of Western blots evaluating BiP expression after 6-h treatments with α-mangostin in LNCaP cells. (**C**) Immunoprecipitation performed in LNCaP cells after 24-h treatments of 10 µM α-mangostin. The AR-protein and BiP-protein that were pulled down were both analyzed for the presence of AR and BiP. (**D**) 22Rν1 cells were transfected with 15 μg of BiP + GST cDNA. After 24 h, cells were treated with 10 μM α-mangostin and both AR-protein and BiP + GST protein were immunoprecipitated and analyzed for the presence of AR or GST. (**E**) SPR analysis of the His-GST-BiP protein revealed that α-mangostin bound to BiP. (**F**) BiP plasmid containing a C41A mutation (15 μg) was transfected into 22Rν1 cells. Immunoprecipitation analyses were repeated, and lysates were analyzed for the presence of AR or myc. * *p* ≤ 0.05. The uncropped blots are shown in File S1.

In PCa cells, BiP is endogenously expressed at low to moderate levels, as we have shown previously [22,23,32]. However, after 24-h treatments of α-mangostin, it was observed that the expressions of PERK and BiP increased dose-dependently in LNCaP, 22Rν1, and VCaP cells treated with α-mangostin (Figure 4A). Additionally, after only 6 h of α-mangostin treatment, the expression of BiP significantly increased in LNCaP cells, demonstrating how quickly α-mangostin begins to affect PCa cells (Figure 4B). Immunoprecipitation assays were carried out in both LNCaP and 22Rν1 cells. After 24-h treatments, LNCaP cell lysates were extracted and incubated with beads conjugated with either AR or BiP. In both cases, the lysates were probed for AR and BiP, and the results revealed that the α-mangostin-treated lysates incubated with AR-conjugated beads contained BiP, while those incubated with BiP-conjugated beads contained AR (Figure 4C). Additionally, a plasmid containing BiP and GST (BiP+GST) was transiently transfected into 22Rν1 cells that were then also treated with α-mangostin for 24 h. Cell lysates were collected and subjected to the same immunoprecipitation procedure, wherein the results revealed the same trend: the α-mangostin-treated lysates incubated with AR-conjugated beads contained BiP+GST (detected at ~100 kDa with a GST antibody), and those incubated with GST-conjugated beads contained AR (Figure 4D).

A Biacore T200 SPR instrument was utilized to evaluate if α-mangostin directly binds to BiP recombinant protein. Using His-capture followed by standard amine coupling, recombinant His-GST-BiP (BiP-N-His-GST) protein was immobilized on an NTA chip. Mangosteen xanthones, including α-mangostin, were monitored in real time for binding interactions. α-Mangostin was found to bind to the BiP recombinant protein with a *K*_D_ calculated to be 21.7 μM (Figure 4E). Using site-directed mutagenesis, a C41A mutation was made in a BiP-myc plasmid. 22Rν1 cells were transfected with the BiPΔC41A plasmid and treated with α-mangostin. Cell lysates were collected and an immunoprecipitation was performed using an AR antibody conjugated to beads or a myc antibody conjugated to beads. Even with mutant BiP, it appeared that α-mangostin promoted an interaction between AR and BiPΔC41A (Figure 4F).

### 3.5. α-Mangostin Is More Effective than Bicalutamide at Slowing Tumor Growth and Cancer Growth in a 22Rν1 Xenograft Study

**Figure 5:**
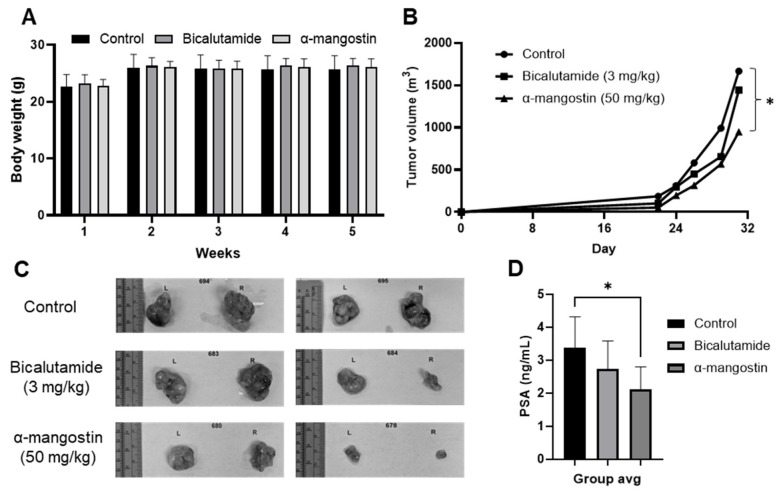
α-Mangostin is more effective Than bicalutamide at reducing tumor size and slowing cancer growth in a 22Rν1 xenograft study. (**A**) The body weights of three cohorts (control, bicalutamide, or α-mangostin) were measured weekly for the duration of the study. (**B**) Tumor volume measurements were taken five times during the study using a digital caliper. (**C**) After the animal sacrifice, mouse tumors were photographed. (**D**) Mouse plasma was collected 1 h after dosing on the day of sacrifice. 50 μL of either the PSA standard or plasma sample was added to a pre-treated ELISA plate, and absorbance readings were taken. * *p* ≤ 0.05.

A xenograft experiment was conducted in male nude athymic mice with 22Rν1 cells, and mice were dosed with either corn oil, bicalutamide in corn oil, or α-mangostin in corn oil. Tumors began to appear about 16 days after implantation, and measurements were first taken on day 22 and last taken on day 31, right before animal sacrifice and tissue collection. The animals’ body weights were recorded weekly for the duration of the study and showed no major or unexpected changes (Figure 5A). The average tumor volumes were determined for each group (on day 31, control = 1668 m^3^, bicalutamide = 1442 m^3^, and α-mangostin = 947 m^3^) and revealed that the α-mangostin group developed significantly smaller tumors compared to the control group (*p* = 0.045) (Figure 5B,C). Mice receiving bicalutamide had smaller tumors, which suggested a slight downward trend in tumor size; however, the effect of α-mangostin was superior when compared to the control. Using a human PSA ELISA kit, the level of PSA in mouse serum was found to be significantly decreased in the α-mangostin group (*p* = 0.05) compared to the control group but not in the bicalutamide group (Figure 5D).

### 3.6. α-Mangostin Decreased AR and AR-V7 Protein Expression and Related Genes In Vivo

**Figure 6:**
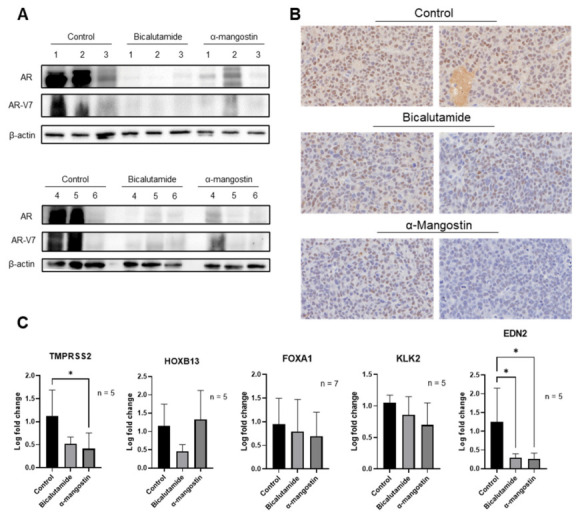
α-Mangostin decreased AR and AR-V7 protein expression and related genes in vivo. (**A**) 20 mg tumor tissue was homogenized in T-PER buffer and protein was extracted. 15 μg protein was loaded, and AR and AR-V7 expressions were detected via Western blot. (**B**) Immunohistochemistry was performed on 30 μg tumor tissue samples, and an AR antibody was used to stain the slides. (**C**) The expression of AR and AR-V7-regulated genes was evaluated in RNA extracted from tumor tissues through qPCR. * *p* ≤ 0.05. The uncropped blots are shown in File S1.

Extracted protein from tumor tissues was analyzed for AR and AR-V7 expression via Western blots. This revealed that there was considerable overexpression of both AR and AR-V7 in the control group, while there were decreases of AR and AR-V7 in the bicalutamide and α-mangostin groups, and in some cases even total depletion (Figure 6A). Routine immunohistochemistry was also performed on tumor tissues and was stained with an AR antibody, showing that there were decreases in the expression of AR in the bicalutamide and α-mangostin groups (Figure 6B). RNA was extracted from tumor tissues, and qPCR analyses of AR and AR-V7 regulated genes revealed that there were significant decreases in TMPRSS2 and EDN2 expressions in the bicalutamide and α-mangostin groups (Figure 6C).

## 4. Discussion

Much effort has been put into developing pharmacotherapies that delay PCa initiation or slow its progression by disrupting AR and the androgen signaling pathway [33]. While anti-androgens remain the current standard of care for primary PCa cases, their clinical effectiveness is limited in androgen-independent cancers, and their long-term usage can result in drug resistance, or CRPC, of which there is only a 30% survival rate [34,35]. Clinically, CRPC is characterized by several mechanisms of resistance to anti-androgens that include overexpression of AR, AR independence, or no AR expression, point mutations in AR, or splice variants of AR, including AR-V7 [36,37]. Resistance to anti-androgens can develop, and they can become ineffective at disrupting androgen signaling, therefore, novel strategies are needed to target mutant AR and AR-V7. In the present study, α-mangostin, a xanthone from the purple mangosteen fruit, was identified as a molecule that can degrade AR, mutant AR, and AR-V7 in both in vitro and in vivo PCa models.

Xanthones from *Garcinia mangostana* and its extracts have been characterized as having broad anti-cancer activity against multiple different cancer cell lines; however, the mechanisms of action behind this remain largely unknown. In the PCa cell lines LNCaP and 22Rν1, α-mangostin decreased cell viability at low micromolar concentrations (Figure 1B). In VCaP cells, α-mangostin still decreased cell viability but was not as effective, showing that α-mangostin is not as effective in cell lines that have an overexpression of AR. Compared to previous reports as well as our own analysis, we have observed α-mangostin to display lower micromolar pharmacological activity relative to well-known phytochemicals evaluated for prostate cancer, including EGCG, diindolylmethane (DIM), and genistein [21,38,39]. It was also observed that α-mangostin promoted apoptosis in PCa cell lines at concentrations between 0–10 μM, given that α-mangostin dose-dependently increased the levels of cleaved caspase-3 (Figure 1C). This corresponds to the decrease in cell viability that was observed at similar concentrations in the MTT assay. Based on these preliminary results, mechanistic studies were carried out using ≤20 μM concentrations of α-mangostin in order to identify a mechanism or target behind the apoptotic effects observed.

One of the main molecular events involved in PCa cell proliferation is the transcription of androgen-regulated genes, which is promoted by AR and initiated after AR dimerization, nuclear translocation of AR, and binding of androgen response elements to AR [5,7]. We evaluated nuclear translocation of AR in LNCaP, 22Rν1, and VCaP cells and found it to be inhibited after α-mangostin treatments (Figure 1D and Figure S1), indicating that transcription of AR-regulated genes may be slowed in response to xanthone treatment. Out of the ~800 different genes regulated by AR, various genes have been found to play more essential roles in promoting carcinogenesis or have been linked to having a poorer PCa prognosis. FOXA1, KLK2, and TMPRSS2 gene expressions were all significantly inhibited by α-mangostin, confirming that nuclear translocation of AR is occurring and that PCa cell proliferation is being affected (Figure 1E). KLK2, one of the genes whose expression was most significantly decreased by α-mangostin, plays a role in carcinogenesis and tumor metastasis, and it was recently revealed that high KLK2 expression correlates with PCa severity and aggressiveness [40,41]. This has been shown to be especially true in TMPRSS2-ERG negative PCa; TMPRSS2 is well characterized as an AR-responsive and prostate-specific gene that can mutate and form TMPRSS2-ERG, one of the most common mutations in PCa that can be used as a prognostic marker [42,43]. Since α-mangostin decreases the expression of both of those genes, this may be one of the mechanisms by which α-mangostin decreases PCa cell proliferation.

Interestingly, the nuclear translocation of AR-V7 was also inhibited, and the expressions of AR-V7-regulated genes, including HOXB13 and EDN2, significantly decreased after α-mangostin treatments (Figure 1C,D). AR-V7 functions independently of ligand binding and drives PCa even after the cells lose dependence on full-length AR and is reported to promote gene transcription through non-canonical nuclear import and signaling [44]. Though the relationship between HOXB13 and AR is controversial, a strong connection between HOXB13 and AR-V7 has been reported, showing that HOXB13 binds to AR-V7 and contributes to its oncogenic function [45,46]. EDN2 has been identified as one of the few downstream targets that are unique to AR-V7; it is reported to be induced by AR-V7 but repressed by AR and is known to regulate vasculature development and autocrine survival [47,48]. The decrease seen in the expression of EDN2 after α-mangostin treatments may have larger implications for the antitumor activity of α-mangostin and one possibility is that it could disrupt in vivo processes including angiogenesis. Additionally, though typically associated with AR, FOXA1 was recently reported to be differentially regulated by both AR and AR-V7, suggesting that FOXA1 could be a key target in CRPC and bringing importance to the ability of α-mangostin to decrease FOXA1 gene expression [49]. Overall, it was a significant finding that AR-V7, a mutant form of AR that has yet to be targeted, and AR-V7-regulated genes were affected by α-mangostin.

Clinically, the effectiveness of PCa drugs on cancer progression can be evaluated by measuring PSA levels or, more recently, by evaluating or genotyping the AR protein that is present [50,51,52]. Given the important role of AR in PCa and its prominence in the PCa drug discovery pipeline, we prioritized AR in some of our initial mechanistic in vitro studies. Interestingly, while most anti-androgens bind to AR and block downstream transcription, we observed that the expression of AR itself decreased after α-mangostin treatments (Figure 2A). While AR is normally overexpressed in PCa, these cell lines all have varying expressions of AR and varying dependence on or sensitivity to AR, so it was significant that α-mangostin overcame this and not only decreased cell viability but also decreased the expression of AR in all three cell lines [53]. Even more unique was the finding that α-mangostin dose-dependently decreased the expression of AR-V7 in 22Rν1 and VCaP cells (Figure 2A,B). This was one of our most significant findings, as AR-V7 has been found to be expressed in up to ~75% of patients who have already received androgen deprivation therapy and represents an important obstacle to the successful treatment of PCa with currently approved drugs [54,55]. Through immunoprecipitation assays, we confirmed that α-mangostin was not just inhibiting the translation of AR and AR-V7, but that it was also promoting the ubiquitination and degradation of AR and AR-V7 (Figure 2C). We confirmed that degradation was being promoted and that it was occurring through the proteasome by co-treating cells with α-mangostin and either a proteasome inhibitor, MG-132, or a proteasome activator, PD169316. In cells treated with MG-132 and α-mangostin, the normal decrease in AR expression was reversed, whereas in cells treated with PD169316 and α-mangostin, the normal decrease in AR expression was exaggerated, and AR’s expression was almost completely ablated (Figure 2D,E). Degradation of AR and/or AR-V7 shuts down the androgen signaling pathway in PCa cells and may even be responsible for the observed decrease in cell viability of PCa cells.

Our data also shows that α-mangostin also decreases the expression of AR containing either E256K, T818D, or T878A mutations (Figure 3B), which is significant because point mutations are also a common mechanism of drug resistance in PCa [56,57]. We determined that the nuclear translocation of ARΔT878A is also inhibited by α-mangostin (Figure 3C). The decrease in mutant AR expression was most apparent with ARΔT878A, which was an important finding as the T878A mutation is well documented to promote resistance to abiraterone [57]. While further confirmation is required to determine if AR is actually degrading these forms of mutant AR, this would be consistent with the degradation of AR and AR-V7 that α-mangostin promotes. Previous attempts to develop molecules that degrade AR have been largely unsuccessful at degrading both AR and AR with point mutations or AR-V7, and it is a significant finding that α-mangostin appears to degrade all three [58,59,60]. These data suggest that α-mangostin likely does not exert its activity by binding to the ligand binding domain of AR, as anti-androgens do, and we hypothesize that α-mangostin overcomes this by upregulating and binding to BiP.

Previously, our lab has published results characterizing the role of the unfolded protein response pathway (UPR) in PCa cells that have been treated with various mangosteen xanthones or extracts [22,23,29], so we suspected that parts of this pathway would be likely targets of α-mangostin, too. We explored the possibility that α-mangostin was promoting a protein-protein interaction between AR and BiP after seeing that there was a significant increase in the expression of BiP in treated cells (Figure 3D and Figure 4A,B). This was consistent with our previous findings and aligned with our hypothesis that AR was being degraded, since degradation is a common downstream mechanism used by the UPR to alleviate cell stress or an imbalance in proteostasis [61]. Through immunoprecipitation assays, we observed that α-mangostin promoted an interaction between AR and BiP (Figure 4C) and that this interaction was also promoted in 22Rν1 cells treated with α-mangostin that had been transfected with exogenous BiP that contained a His-GST tag (Figure 4D). As BiP is a cell stress and chaperone protein, we hypothesize that α-mangostin increases the expression of BiP, which then associates with AR and chaperones it to the proteasome for ubiquitination and degradation [62]. Our previous data showed that there was no change in the expression of UPR proteins after α-mangostin treatments in primary prostate epithelial cells from PCa patients, indicating that this unique protein-protein interaction only occurs in PCa cells and only when they are treated with α-mangostin [23].

We next focused on elucidating key residues that may help facilitate the protein-protein interaction between AR and BiP. One possibility was that α-mangostin could be binding to the ATPase domain of BiP, which would be consistent with reports of other small molecules binding to BiP [63,64]. However, considering that the basal ATPase activity of BiP is relatively low, it proved difficult to assess changes in ATPase activity without a more sensitive technique (Figure S2). This would also nullify our data showing that BiP is activated and available to chaperone AR after α-mangostin treatments, since BiP requires ATPase activity to interact with a client protein [65,66]. Ultimately, SPR was selected to assess this small molecule–protein interaction and confirmed that α-mangostin does indeed bind to BiP (Figure 4E). Without any further information about this interaction, we postulated that α-mangostin could be binding to one of the two cysteine residues on BiP’s structure and forming a quinone.

It has previously been hypothesized that natural products can oxidize during in vitro metabolism and are able to form quinones [67,68]. Even more interesting is that quinones have been documented to promote anticancer activity [67,69,70]. Considering the prevalence of hydroxyl groups on many mangosteen xanthones structures’, with three hydroxyl groups present on α-mangostin, it could be that a hydroxyl is oxidized and becomes available to bind to one of the two cysteine residues on BiP, which readily bind with quinones [71]. To evaluate this, site-directed mutagenesis was performed on the BiP plasmid to mutate the cysteine at residue 41 in BiP’s sequence to an alanine (C41A), which would not preferentially bind to a quinone. The BiPΔC41A plasmid was then transfected into 22Rν1 cells, which were treated with α-mangostin, and then analyzed for the presence of AR-BiP protein-protein interaction through a co-immunoprecipitation (Figure 4F). Though this preliminary data shows that cysteine 41 was not essential to promote this protein-protein interaction, it may be possible that the second cysteine residue on BiP (C419) could promote this binding. Future studies using proteomic approaches would be useful to understand if the AR-BiP protein complex that occurs after α-mangostin treatment in PCa cells contains α-mangostin, which would give rise to the thought that α-mangostin itself may be binding to BiP and activating BiP to promote AR degradation.

Finally, we evaluated the in vivo efficacy of α-mangostin in an athymic nude mouse model to determine its ability to degrade AR and AR-V7 in vivo. We selected 22Rν1 cells so the model would be representative of a CRPC environment and to be able to determine any effects on AR-V7. Athymic nude male mice were xenografted and dosed daily with either control, bicalutamide, or α-mangostin through oral gavage for 31 days. All treatments appeared to be safe and well tolerated (Figure 5A), and we have previously reported that α-mangostin is orally bioavailable and have described its respective pharmacokinetic profile in mice; furthermore, alpha-mangostin was shown to be safe and well tolerated when evaluating complete blood chemistry (CBC) [24]. At the conclusion of this study, mice in the α-mangostin group had tumors that were ~40% smaller than those of mice in the control or bicalutamide groups (Figure 5B,C). It appeared that these tumors were not only significantly smaller but also grew more slowly than tumors from mice in the control and bicalutamide groups. We detected a significant decrease in PSA levels in plasma from mice in the α-mangostin group through an ELISA and observed that these mice had PSA levels of ~2.0 ng/mL, as opposed to ~4.0 ng/mL, which clinically would be seen as promising progress following a treatment (Figure 5D) [72,73].

Furthermore, our analysis of AR and AR-V7 expressions in tumor tissue samples from the α-mangostin group showed that there were decreases in protein levels and almost complete ablation in some cases (Figure 6A,B). While this same trend was apparent in tumor tissue samples from the bicalutamide group, α-mangostin was the only treatment that overcame this CRPC-like mouse model and yielded significant decreases in cancer growth. A follow-up experiment evaluating the expression of AR after either α-mangostin or bicalutamide treatments in 22Rν1 cells suggested that the in vitro and in vivo results diverge in terms of the expressions of AR and AR-V7 after bicalutamide treatment (Figure S3). This discrepancy may be explained by the fact that bicalutamide did show a small amount of efficacy in reducing tumor size and PSA levels, indicative of PCa progression slowing and therefore androgen signaling decreasing; however, further studies should be conducted to provide a more clear explanation. There were also decreases seen in the expression of AR- and AR-V7-regulated genes in RNA extracted from the tumor tissues, though only the decreases in TMPRSS2 and EDN2 were significant (Figure 6C). It could be speculated that the decreases seen in EDN2 could have played a role in the antitumor effects that bicalutamide and α-mangostin had and that there may have been a decrease in vasculature within these tumors [47]. The present study should also be expanded in the future to be able to determine if the observed decreases in AR and AR-V7 protein levels in the α-mangostin group are indicative of in vivo degradation, which would provide data consistent with our proposed in vitro mechanism.

## 5. Conclusions

These findings are consistent with previous reports of the anticancer and antitumor activities of both α-mangostin and mangosteen fruit extract [21], and future studies using either combination approaches or other mangosteen xanthones are suggested to understand the full anticancer potential of the purple mangosteen. In conclusion, these results highlight the ability of α-mangostin to target wild-type and mutant AR for degradation through the promotion of the chaperone protein BiP. This represents a novel approach to targeting the androgen signaling pathway in PCa and appears to be effective in both in vitro and in vivo PCa models.

## Data Availability

The data presented in this study are available within this article and its Supplementary Materials.

## Supplementary Materials

The following supporting information can be downloaded at: https://www.mdpi.com/article/10.3390/cancers15072118/s1, Figure S1: Nuclear translocation of AR and AR-V7 in VCaP cells; Figure S2: ATPase activity of recombinant His-GST-BiP protein; Figure S3: Bicalutamide does not decrease AR or AR-V7 expression in vitro. File S1: Western blots originals.
