# Supplementary material for: α-Mangostin Promotes In Vitro and In Vivo Degradation of Androgen Receptor and AR-V7 Splice Variant in Prostate Cancer Cells
Source: Cancers (Basel). 2023 Apr 1;15(7):2118. doi: 10.3390/cancers15072118 (PMC10093438; doi:10.3390/cancers15072118)
Supplement: Supplementary file 1 [file cancers-15-02118-s001.zip › File S1 Western blots originals.pptx]

## Slide 1
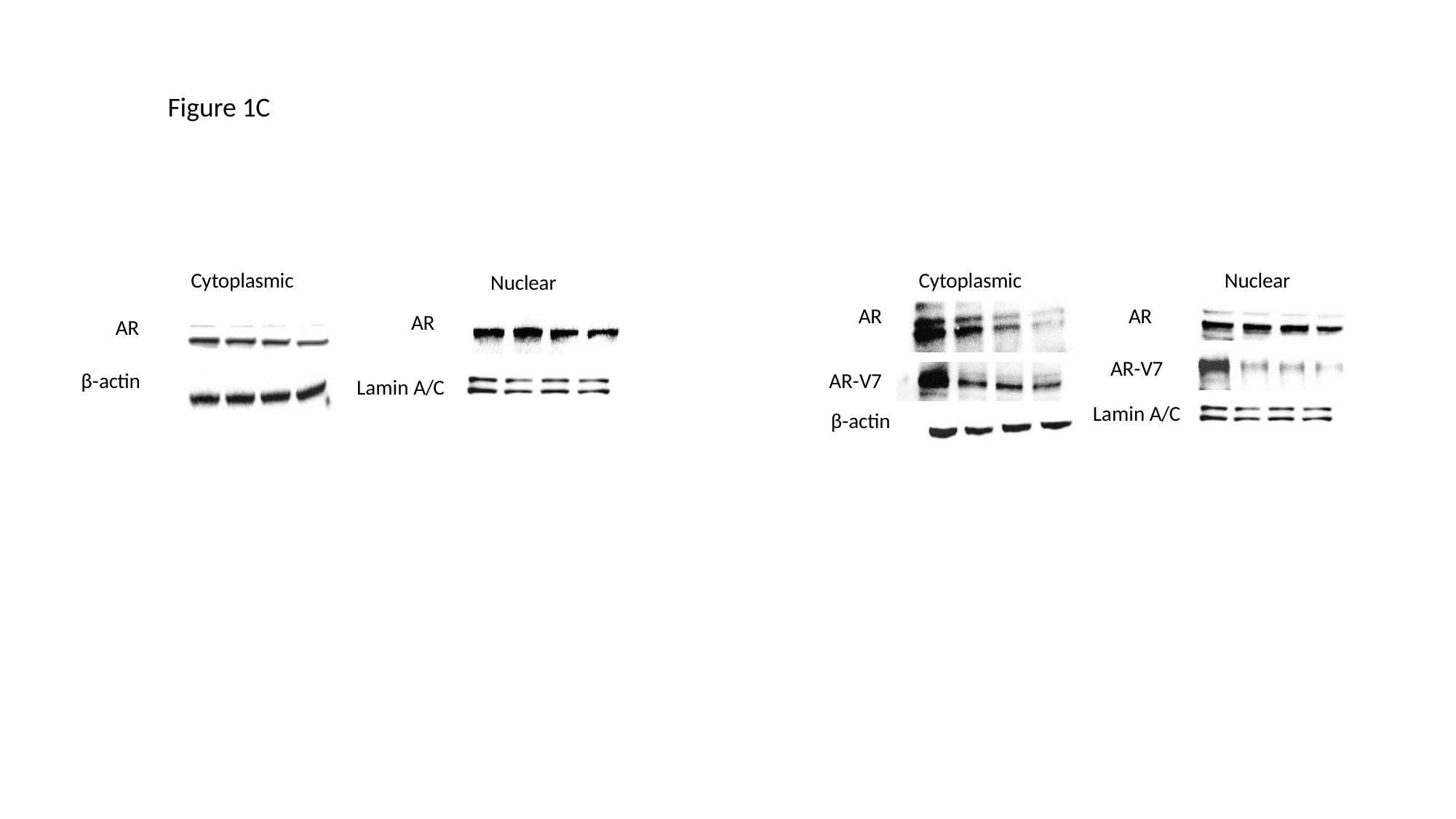

# Figure 1C
Cytoplasmic
Cytoplasmic
Nuclear
Nuclear
AR
AR
AR
AR
AR-V7
β-actin
AR-V7
Lamin A/C
β-actin
Lamin A/C

## Slide 2
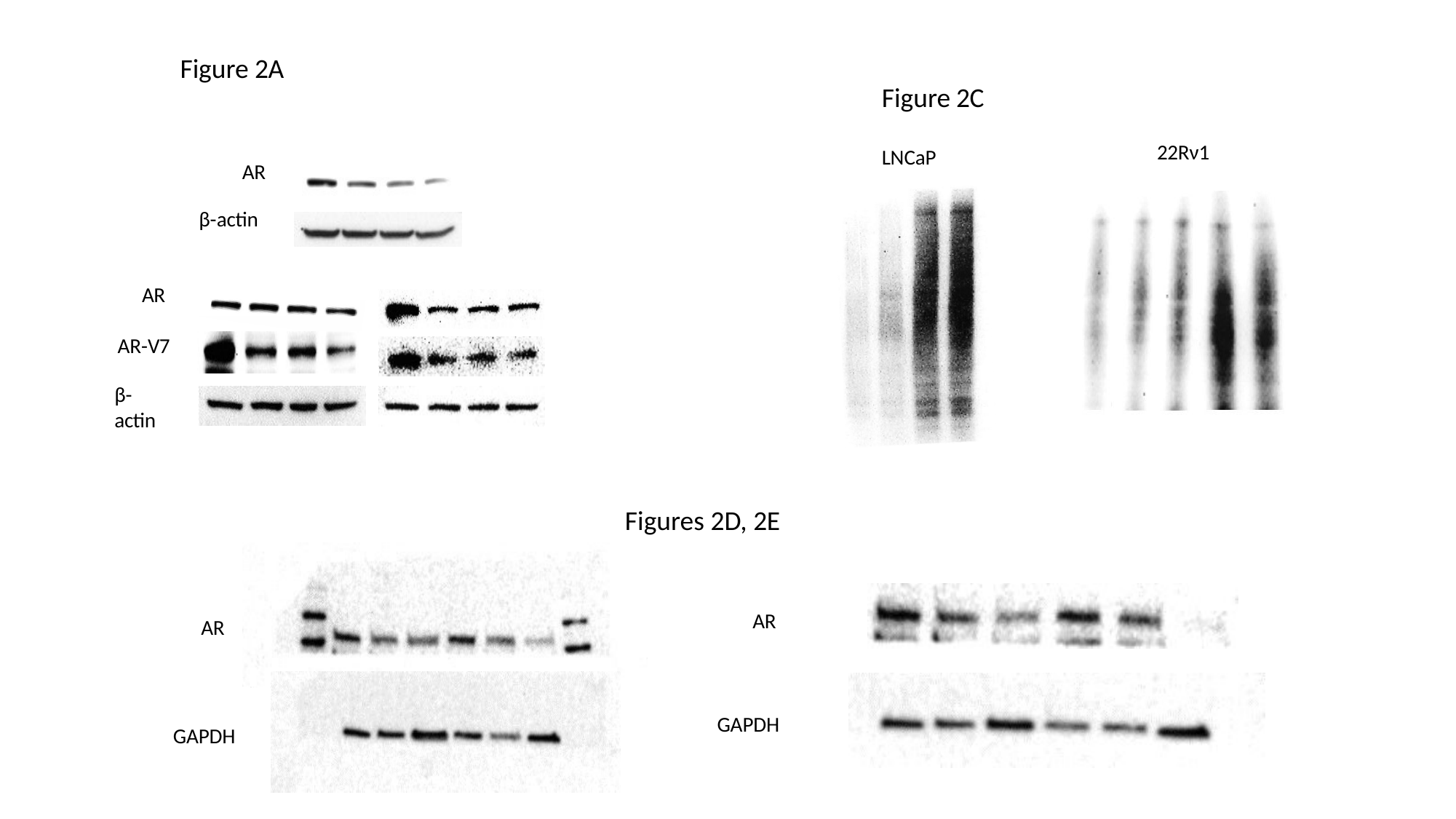

# Figure 2A
Figure 2C
22Rν1
LNCaP
AR
β-actin
AR
AR-V7
β-actin
Figures 2D, 2E
AR
AR
GAPDH
GAPDH

## Slide 3
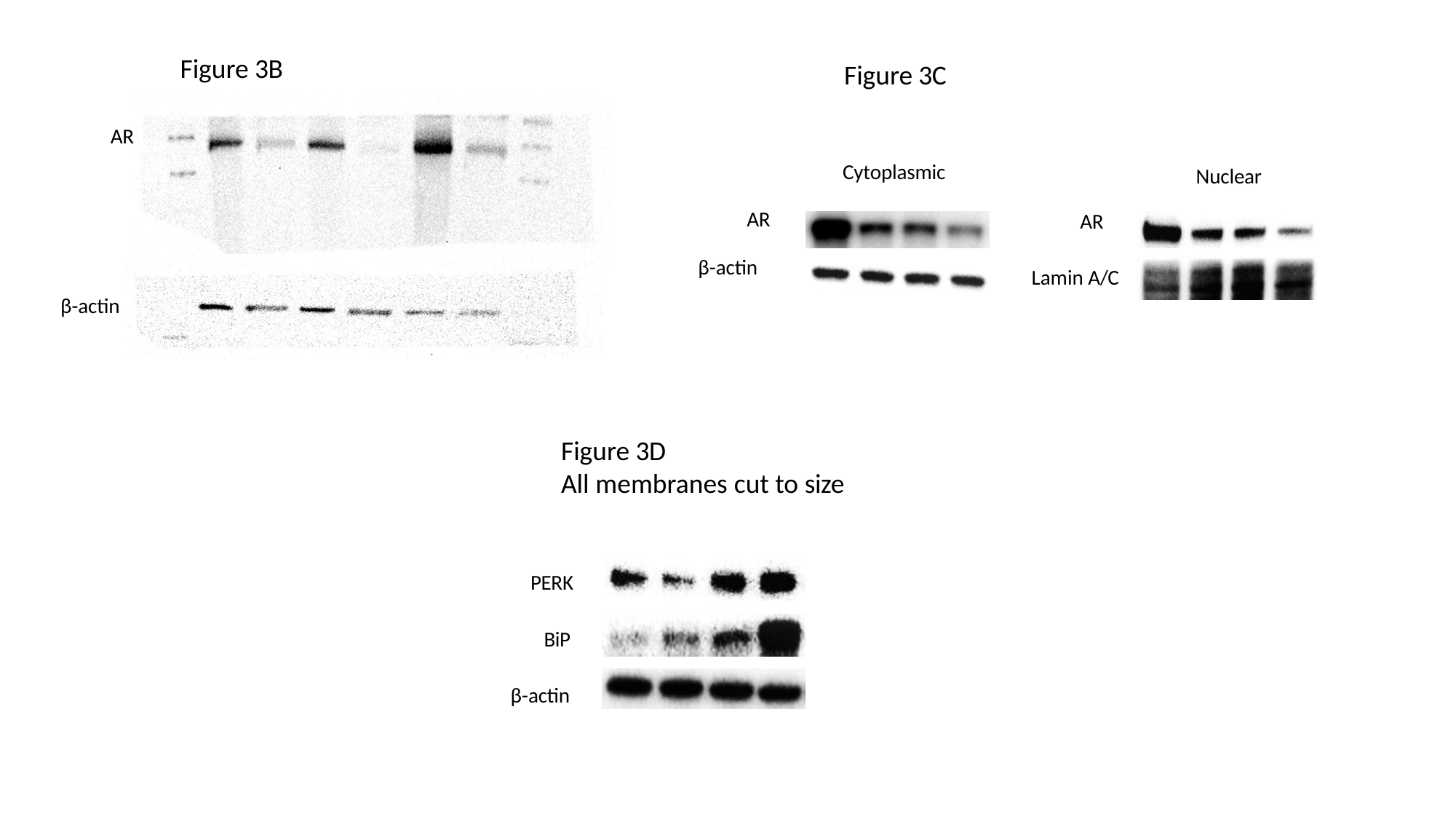

# Figure 3B
Figure 3C
AR
Cytoplasmic
Nuclear
AR
AR
β-actin
Lamin A/C
β-actin
Figure 3D
All membranes cut to size
PERK
BiP β-actin

## Slide 4
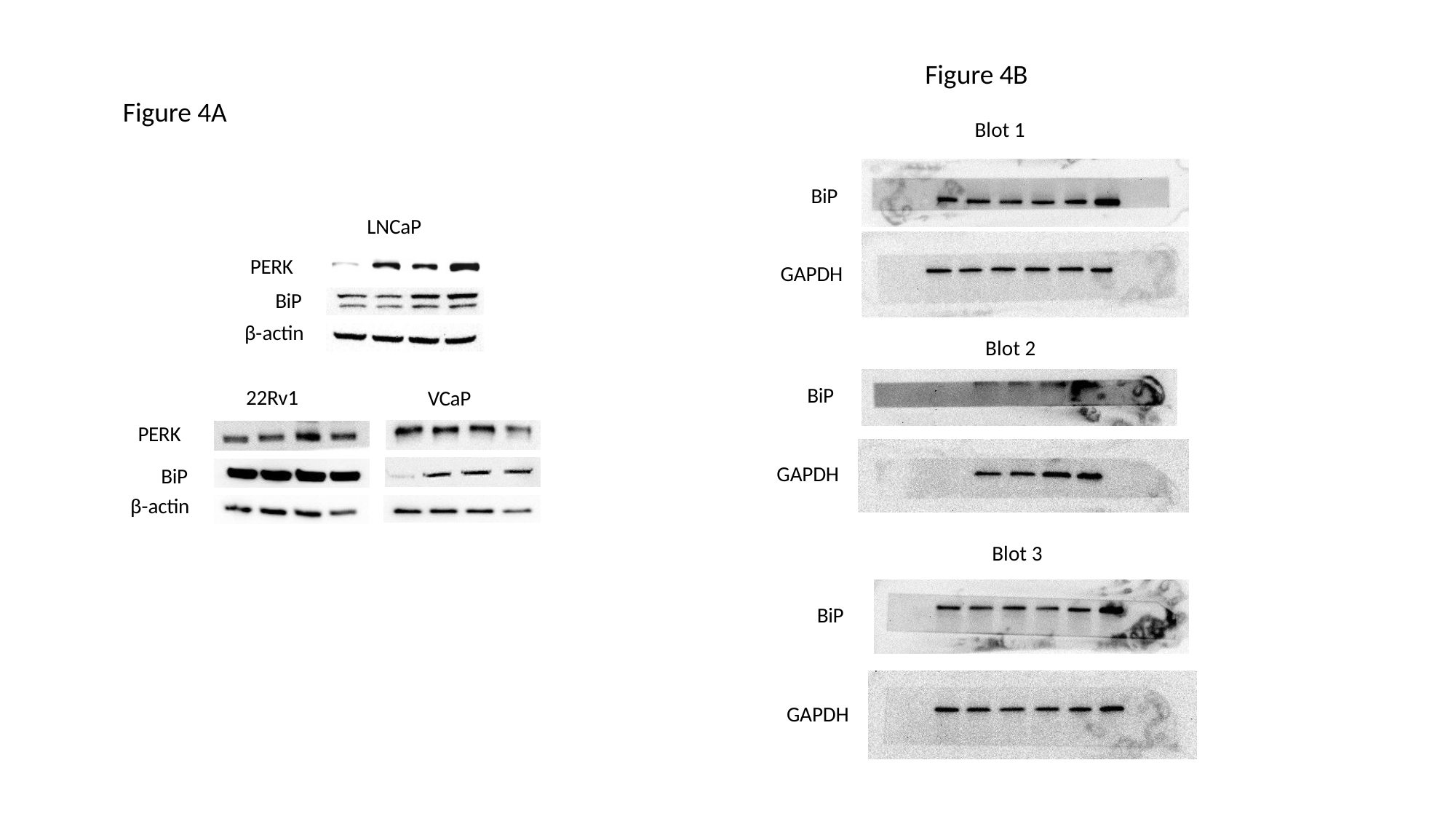

# Figure 4B
Figure 4A
Blot 1
BiP
LNCaP
PERK
BiP β-actin
GAPDH
Blot 2
BiP
22Rv1
VCaP
PERK
BiP β-actin
GAPDH
Blot 3
BiP
GAPDH

## Slide 5
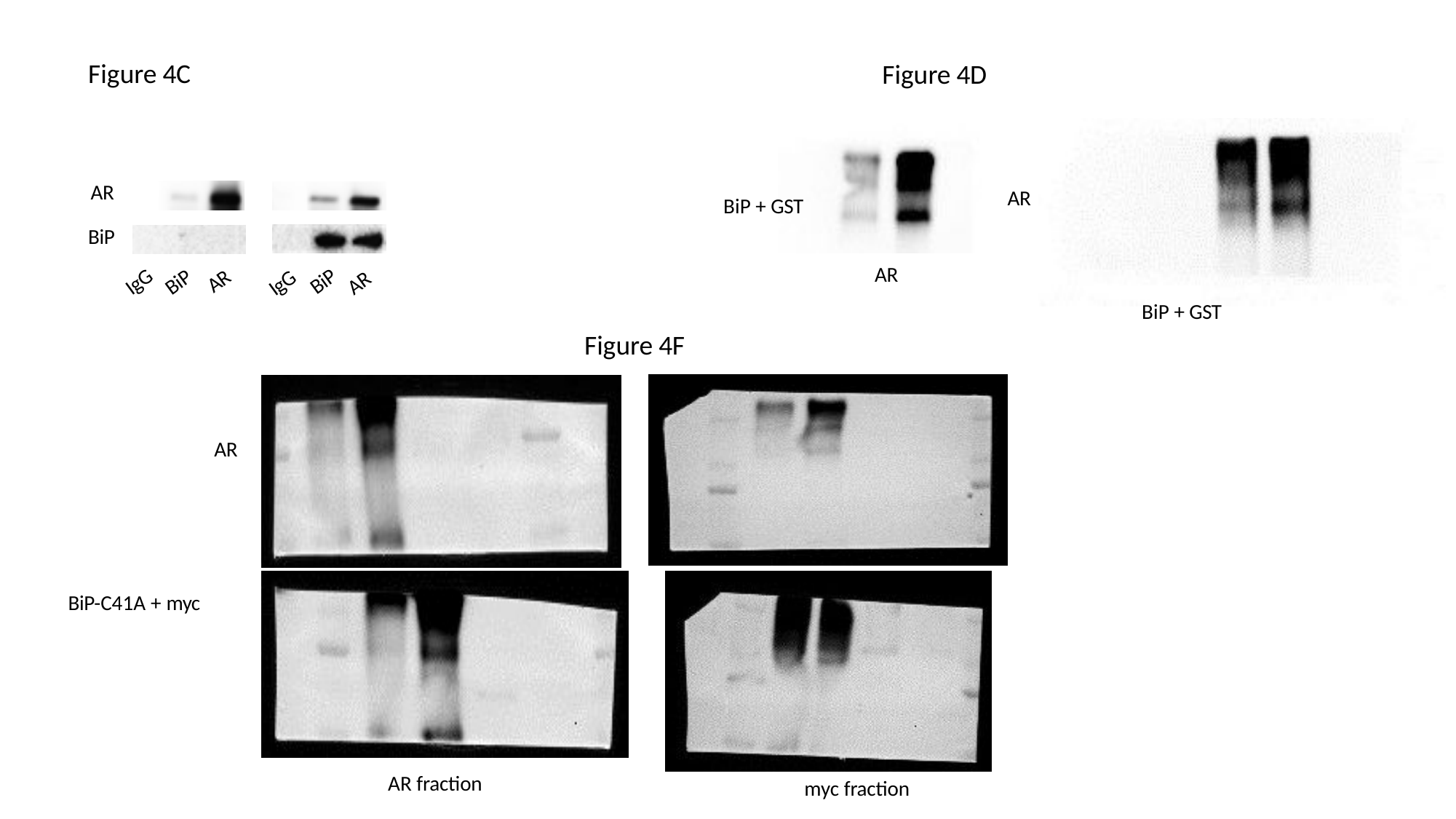

# Figure 4C
Figure 4D
AR
BiP
AR
BiP + GST
AR
AR
BiP
IgG
BiP
AR
IgG
BiP + GST
Figure 4F
AR
BiP-C41A + myc
AR fraction
myc fraction

## Slide 6
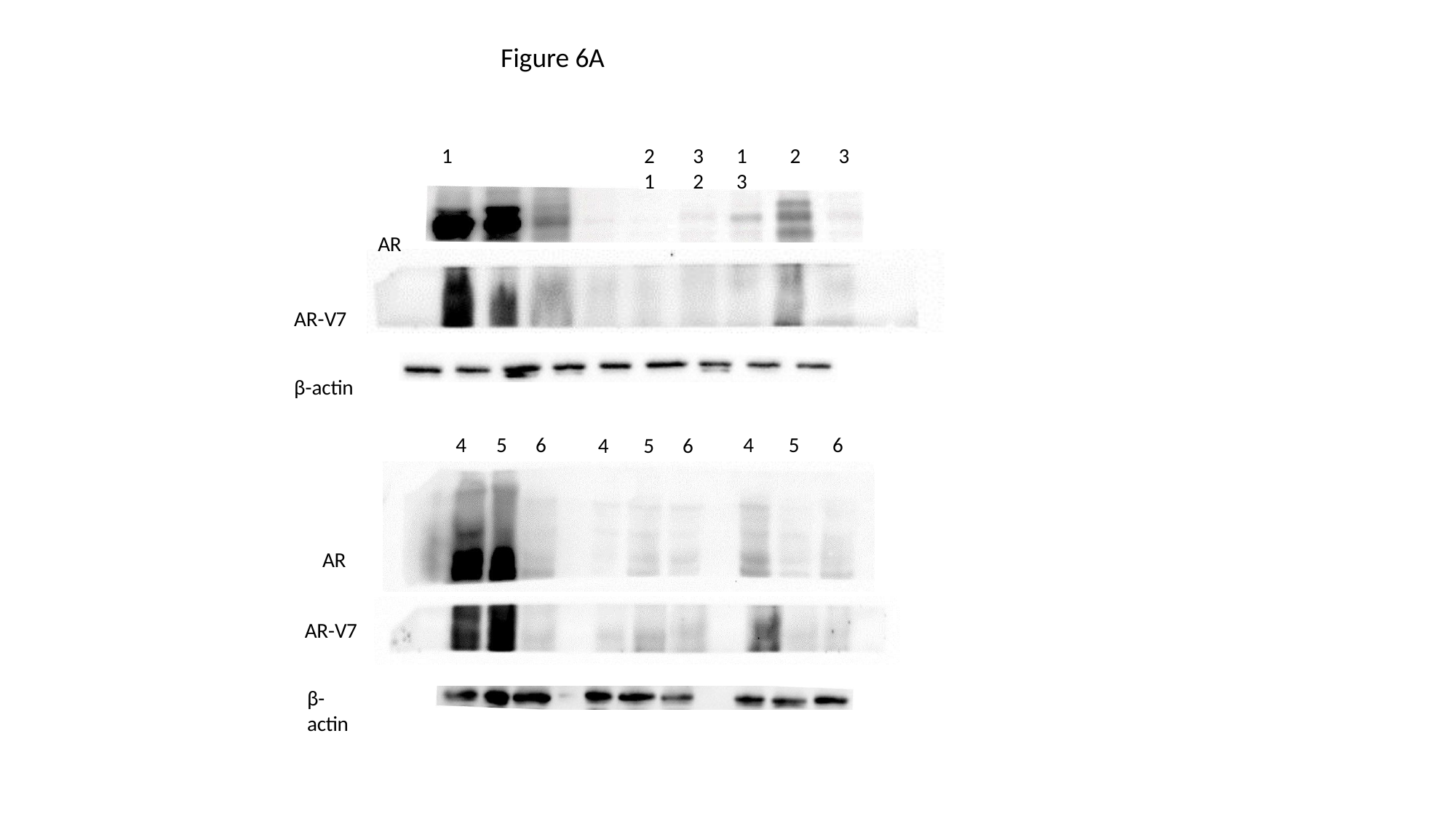

Figure 6A
1	2	3	1	2	3	1	2	3
AR
AR-V7
β-actin
4	5	6
4	5	6
4	5	6
AR
AR-V7
β-actin
