# Supplementary material for: α-Mangostin Promotes In Vitro and In Vivo Degradation of Androgen Receptor and AR-V7 Splice Variant in Prostate Cancer Cells
Source: Cancers (Basel). 2023 Apr 1;15(7):2118. doi: 10.3390/cancers15072118 (PMC10093438; doi:10.3390/cancers15072118)
Supplement: Supplementary file 1 [file cancers-15-02118-s001.zip › Supplementary Figure S2.pdf]

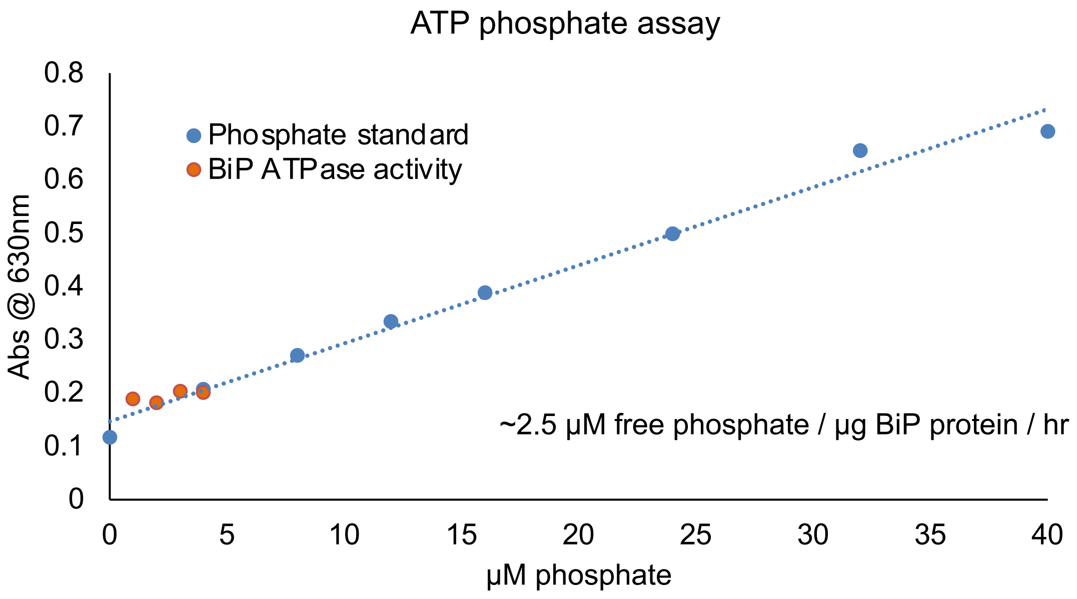

Figure S2. ATPase activity of recombinant His-GST-BiP protein.
The ATPase activity of purified BiP-GST protein was monitored using a Malachite Green Phosphate assay.
