# Supplementary material for: α-Mangostin Promotes In Vitro and In Vivo Degradation of Androgen Receptor and AR-V7 Splice Variant in Prostate Cancer Cells
Source: Cancers (Basel). 2023 Apr 1;15(7):2118. doi: 10.3390/cancers15072118 (PMC10093438; doi:10.3390/cancers15072118)
Supplement: Supplementary file 1 [file cancers-15-02118-s001.zip › Supplementary Figure S3.pdf]

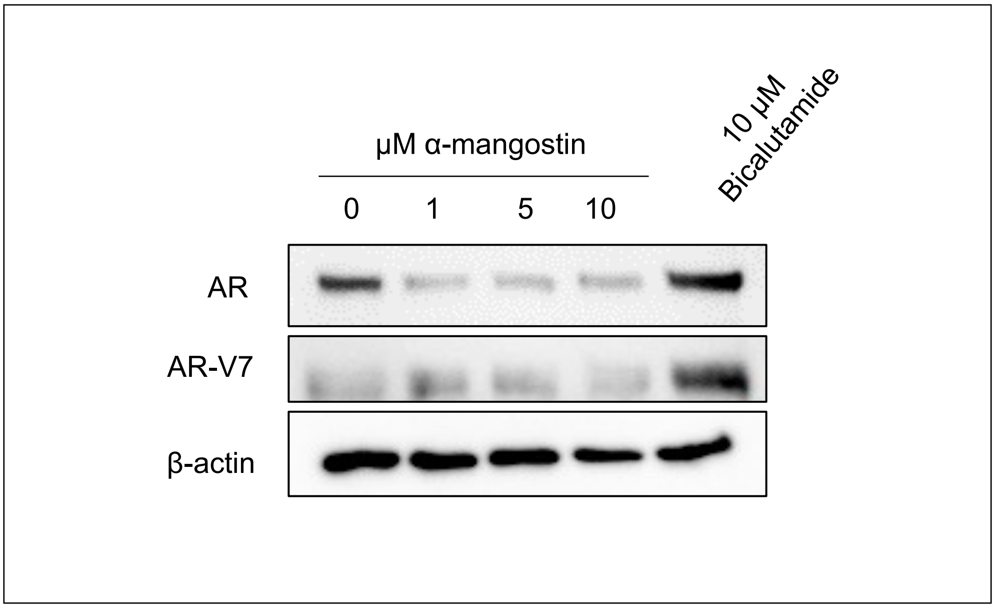

Figure S3. Bicalutamide does not decrease AR or AR-V7 expression *in vitro*.
In 22Rv1 cells, bicalutamide did not decrease the expressions of AR or AR-V7 whereas α-mangostin did.
